# What matters during a hypotensive episode: fluids, vasopressors, or both?

**DOI:** 10.1186/cc10806

**Published:** 2012-03-20

**Authors:** J Lee, R Kothari, JA Ladapo, DJ Scott, LA Celi

**Affiliations:** 1 Massachusetts Institute of Technology, Cambridge, MA, USA; 2 Mount Sinai School of Medicine, New York City, NY, USA; 3 New York University School of Medicine, New York City, NY, USA

## Introduction

The objective of this retrospective study was to investigate the relationships between fluid and vasopressor interventions and patient outcomes. In intensive care, it is imperative to resolve hypotensive episodes (HEs) in a timely manner in order to minimize end-organ damage. The current clinical practice is first to attempt fluid resuscitation and then to follow with vasopressor therapy if fluid resuscitation is unsuccessful. However, the effects of fluid and vasopressor interventions on patient outcomes have not been clearly established.

## Methods

Hypotension was defined as MAP below 60 mmHg. The primary outcome was in-hospital mortality. Secondary outcomes included ICU LOS, HE duration, Hypotension Severity Index (HSI) (MAP curve area below 60 mmHg during the HE), and rise in serum creatinine. The patient cohort included patients in the MIMIC-II database [1] who experienced a single HE. Multivariate logistic regression and propensity score analysis were employed. Sensitivity analyses were conducted in subpopulations stratified by treatment type and diagnosis.

## Results

A total of 3,163 patients in MIMIC-II met the inclusion criteria. The multivariate regression results showed that fluid resuscitation was significantly associated with shorter ICU LOS (OR = 0.71, *P *= 0.007) and greater HSI (OR = 1.26, *P *= 0.04). Vasopressor administration significantly decreased HE duration (OR = 0.29, *P *< 0.001) and HSI (OR = 0.72, *P *= 0.002) but was correlated with increased in-hospital mortality risk (OR = 2.86, *P *< 0.001) (even after propensity adjustment; OR = 2.44, *P *< 0.001), prolonged ICU LOS (OR = 1.29, *P *= 0.04), and rise in serum creatinine (OR = 1.44, *P *= 0.002). Sensitivity analyses in treatment-specific and diagnosis-specific subpopulations corroborated the relationship between vasopressors and increased in-hospital mortality.

## Conclusion

Regarding the relationship between vasopressor therapy and in-hospital mortality, similar findings have been reported in previous studies analyzing sepsis [2], cardiac surgery [3], and heart failure [4]. We speculate that benefits of vasopressor use may be restricted to subsets of patients with specific conditions. This study illustrates the utility of electronic medical records in research when randomized controlled trials are difficult to conduct.
